# αβ T cell receptor germline CDR regions moderate contact with MHC ligands and regulate peptide cross-reactivity

**DOI:** 10.1038/srep35006

**Published:** 2016-10-24

**Authors:** Meriem Attaf, Stephan J. Holland, Istvan Bartok, Julian Dyson

**Affiliations:** 1 Section of Molecular Immunology, Department of Medicine, Imperial College London, Du Cane Road, London, W12 0NN, UK

## Abstract

αβ T cells respond to peptide epitopes presented by major histocompatibility complex (MHC) molecules. The role of T cell receptor (TCR) germline complementarity determining regions (CDR1 and 2) in MHC restriction is not well understood. Here, we examine T cell development, MHC restriction and antigen recognition where germline CDR loop structure has been modified by multiple glycine/alanine substitutions. Surprisingly, loss of germline structure increases TCR engagement with MHC ligands leading to excessive loss of immature thymocytes. MHC restriction is, however, strictly maintained. The peripheral T cell repertoire is affected similarly, exhibiting elevated cross-reactivity to foreign peptides. Our findings are consistent with germline TCR structure optimising T cell cross-reactivity and immunity by moderating engagement with MHC ligands. This strategy may operate alongside co-receptor imposed MHC restriction, freeing germline TCR structure to adopt this novel role in the TCR-MHC interface.

Conventional αβ T cells are MHC ‘restricted’, using their T cell receptors (TCR) to engage with MHC class I and II molecules presenting peptide epitopes. Thymocyte development is dependent on recognition of self-peptides while T cell activation is mostly driven by non-self peptides. Two mechanisms have been proposed to underlie this strong ligand bias. The CD8 and CD4 T cell co-receptors have dual specificity for (i) MHC class I and II respectively and (ii) the intracellular proximal kinase lck^1^. Consequently, only when MHC ligands are engaged will lck be actively co-localised with the TCR/CD3 complex allowing efficient phosphorylation of CD3 ITAM motifs and initiating signal transduction^2^,^3^,^4^,^5^. As the co-receptor binding sites on MHC class I and II are largely conserved across alleles^6^, this indirect mechanism will impose self-MHC restriction without the need for conventional ‘hardwired’ ligand specificity. Alternatively, based on conserved germline CDR residues and recurrent interactions between TCR and the MHC α-helices, specificity for MHC could be ‘hardwired’ and intrinsic to the receptor itself^7^,^8^.

Previously, using retrogenic mice, we (i) redirected *in vivo* VDJ recombination to randomly diversify germline CDR structure and (ii) used chimeric TCR-β chains with γδ lineage germline loops to show that the germline CDRs are not required for recognition MHC ligands^9^. Rather, based on these data, we hypothesised the TCR adopts an antibody-like strategy for engaging MHC ligands, scanning cell surface molecules for interfaces which are compatible with thymic selection. Here, we confirm this antibody-like recognition by exchanging the TCRβ germline CDRs for immunoglobulin (Ig) heavy (H) and light (L) chain germline CDRs which direct thymic selection of both CD4 and CD8 αβ T cell repertoires. These findings suggest that the TCR germline CDRs may play a more subtle role in ‘fine-tuning’ engagement with MHC ligands. To investigate this hypothesis, we produced TCR transgenic strains with structurally ‘simplified’, flexible germline CDRs through multiple glycine and alanine substitutions. Our analyses show that wild-type TCR structures moderate engagement with MHC to optimise T cell selection and peptide cross-reactivity within the peripheral T cell repertoire.

## The TCR Adopts an antibody-like strategy for ligand recognition

**Figure 1:**
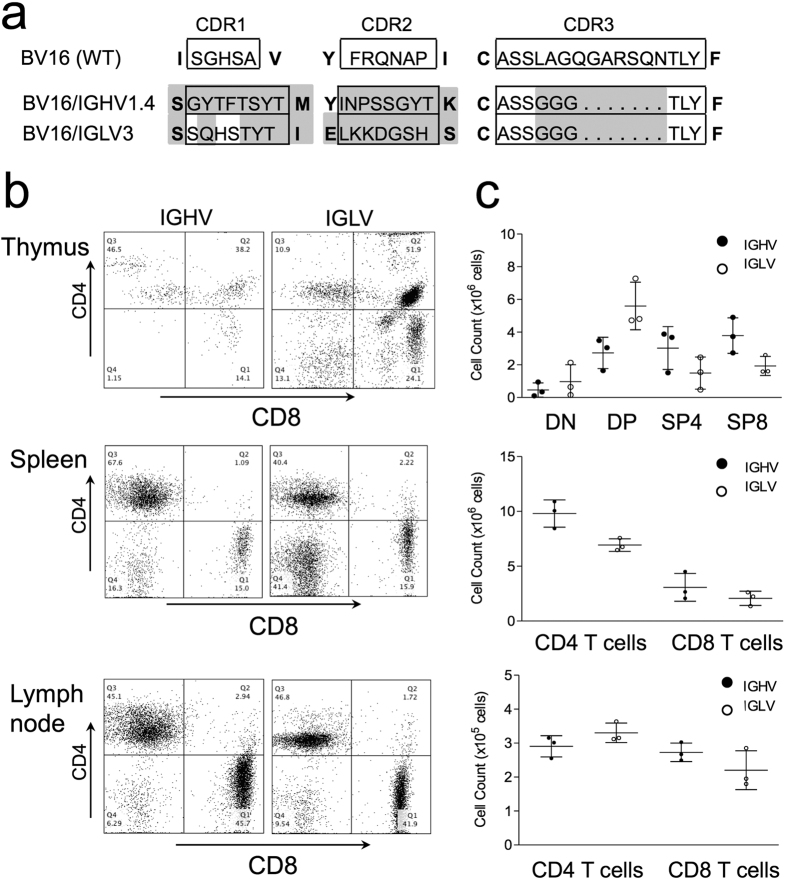
Chimeric TCR-β chains with immunoglobulin germline CDRs direct T cell selection. (**a**) Wild-type BV16 TCR germline CDR1 and 2 regions are shown (top) with corresponding hybrid TCR-β chains containing IgHV and IgLV CDRs regions below. To minimise the role of CDR3 in ligand engagement, it was reduced to a triple glycine in all constructs. For identification, CDRs are boxed. Mutated amino-acids are highlighted. Framework residues are shown in bold. Deleted amino-acids are indicated with dots. (**b**) Retrogenic mice (n = 3 in each group) were produced with the chimeric TCR-β chains. Left panels show example flow cytometry plots of thymus, spleen and lymph node. Right panels show summaries of percentages of thymocyte populations (DN, DP, SP4, SP8) and spleen/lymph node CD4 and CD8 T cells.

**Figure 2:**
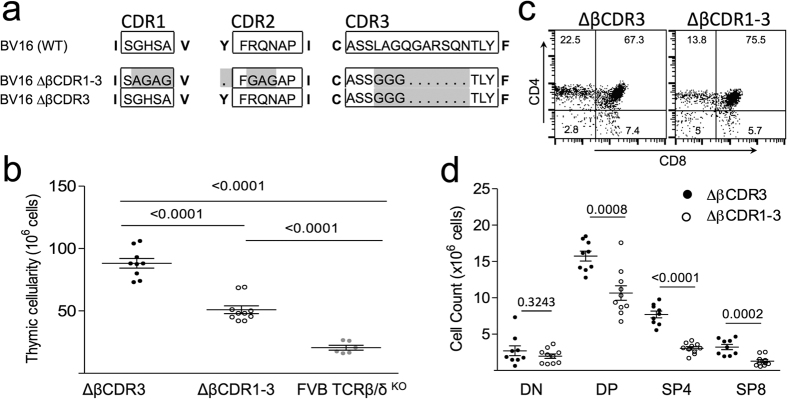
TCR-β mutants and TCR ΔβCDR3 and ΔβCDR1-3 transgenic thymus phenotypes. (**a**) Wild-type BV16 TCR germline CDR1 and 2 regions are shown (top) with ΔβCDR1-3 and ΔβCDR3 TCR-β mutants below. To minimise the role of CDR3 in ligand engagement, it was reduced to triple glycine in both constructs. For identification, CDRs are boxed. Mutated amino-acids are highlighted. Framework residues are shown in bold. Deleted amino-acids are indicated with dots. ΔβCDR1-3 & ΔβCDR3 mutants were used to produce transgenic mice. (**b**) Thymic cellularity of transgenic ΔβCDR3, ΔβCDR1-3 and FVB/N β/δ KO. (**c**) Example of ΔβCDR3 and ΔβCDR1-3 thymic CD4/CD8 profiles. (**d**) Summary of ΔβCDR3 (n = 9) and ΔβCDR1-3 (n = 10) DN, DP, SP4 and SP8 cell numbers.

To directly establish whether the TCR uses an antibody-like strategy of ligand recognition, we produced retrogenic mice expressing chimeric TCR/Ig chains composed of TRBV16 (Vβ11) containing the germline CDR1 and 2 regions from a heavy (H) or light (H) chain immunoglobulin V segment. The IgH and IgL germline CDRs differ in length and composition from TRBV16 and introduce 19 and 15 amino-acid changes respectively (Fig. 1a). To minimise the role of TCR-β CDR3 in ligand engagement, it was reduced to triple glycine in all constructs used in this work (Figs 1a and 2a)^9^,^10^. Retrogenic mice were produced in FVB/N (H2^q^) TCR β/δ KO mice making T cell development dependent on the exogenous chimeric β chain^11^. Both chimeric chains were able to direct the development of mature thymocytes and peripheral T cell compartments (Fig. 1b,c) consistent with the TCR adopting an antibody-like strategy for ligand recognition^9^. Further, these findings support the hypothesis that the CD4 and CD8 co-receptors play a key role in imprinting MHC restriction and suggest that the germline CDR regions may play a secondary role in ‘fine-tuning’ TCR engagement with MHC:peptide ligands.

## Germline TCR structure restrains ligand engagement during thymic T cell development

To explore this idea, we produced a transgenic strain expressing a mutant TRBV16 chain in which germline structure was ‘simplified’ by introducing alanine/glycine substitutions across CDR1 and CDR2 (canonical structure group βCDR1.2/βCDR2.2^12^) (Fig. 2a; ΔβCDR1-3). Additionally, the semi-conserved Y56 at the start of CDR2, which plays a role in controlling MHC engagement^7^, was deleted (Fig. 2a). As for the retrogenic mice, to minimise the role of TCR-β CDR3 in ligand engagement, it was reduced to triple glycine (Fig. 2a). Founder ΔβCDR1-3 and control TRBV16 with mutated CDR3 only (ΔβCDR3; Fig. 2a) transgenic lines were also produced directly in the FVB/N (H2^q^) TCR β/δ KO strain. The remainder of this paper will focus on these two transgenic strains.

**Figure 3:**
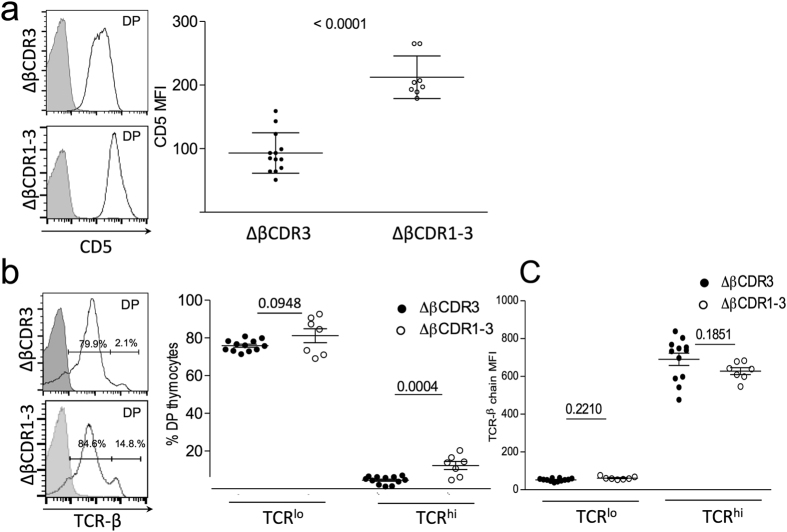
Loss of germline CDR structure increases TCR engagement with thymic ligands. (**a**) Left, example of CD5 expression on ΔβCDR3 and ΔβCDR1-3 DP thymocytes. Right, summary of CD5 mean fluorescence intensity (MFI) on DP thymocytes for ΔβCDR3 (n = 13) and ΔβCDR1-3 (n = 8) transgenic mice. (**b**) Left, example of TCR-β expression on ΔβCDR3 (n = 12) and ΔβCDR1-3 (n = 7) DP thymocytes. Gate indicates TCR^lo^ and TCR^hi^ populations. Right, summary of percentages of TCR^lo^/TCR^hi^ DP thymocytes. (**c**) Summary of TCRβ MFI within the TCR^lo^ and TCR^hi^ gates.

Strikingly, thymic cellularity of the ΔβCDR1-3 transgenic mice was significantly reduced in comparison to the control ΔCDR3 transgenic (Fig. 2b). The numbers of CD4^+^ CD8^+^ double positive (DP) thymocytes and single positive (SP)4 and SP8 thymocytes were also significantly reduced in the ΔβCDR1-3 transgenic (Fig. 2c,d). The ΔβCDR1-3 phenotype is consistent with intensified ligand engagement across the DP pre-selection thymocyte repertoire where the majority of negative selection occurs^13^. This hypothesis was examined by looking at markers of TCR engagement. Expression of the negative regulator of TCR signalling CD5 on DP thymocytes parallels the intensity of interaction between the TCR and MHC^14^ providing a quantitative readout of MHC engagement. CD5 expression on ΔβCDR1-3 DPs was significantly higher than in the control ΔCDR3 transgenic (Fig. 3a). Similarly, up-modulation of the TCR itself is indicative of competence to undergo positive selection^15^. Again, the ΔβCDR1-3 mutant had an increased proportion of TCR^hi^ DP thymocytes in comparison with the ΔCDR3 control transgenic (Fig. 3b). TCR mean fluorescence intensity (MFI) was comparable in TCR^lo^ cells from ΔβCDR3 and ΔβCDR1-3 mice (Fig. 3c). The activation marker CD69 is induced on DP thymocytes undergoing positive or negative selection^16^ and was also expressed on an increased proportion of ΔβCDR1-3 DP thymocytes (Supplementary Fig. S1). We conclude that the TCR repertoire of ΔβCDR1-3 mutant DP thymocytes has enhanced affinity for self-ligands identifying an unexpected role for germline TCR structure in restraining engagement with MHC ligands.

## MHC restriction is imposed independently of germline TCR structure

**Figure 4:**
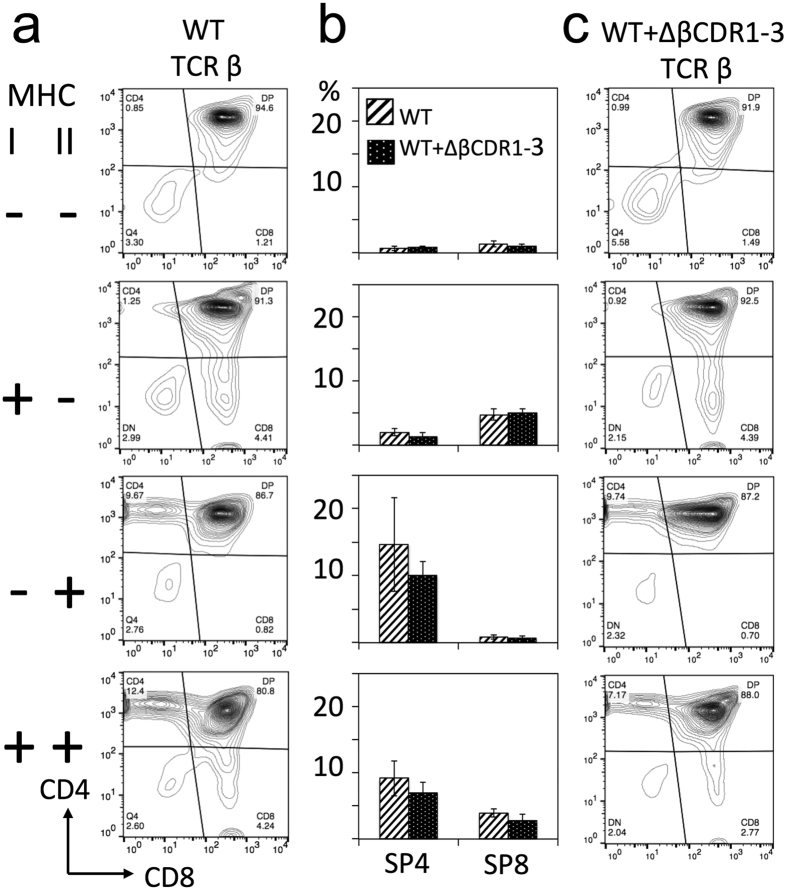
ΔβCDR1-3 expressing thymocytes cells are MHC restricted. Examples of thymic CD4, CD8 profiles of mice with WT TCR (**a**) and WT + ΔβCDR1-3 TCR (**c**). Expression of MHC class I and II are indicated on the left. (**b**) Summary of data from multiple mice (n = 4–16 per group) showing percentages of SP4 and SP8 thymocytes. Gating was set by single stain controls.

**Figure 5:**
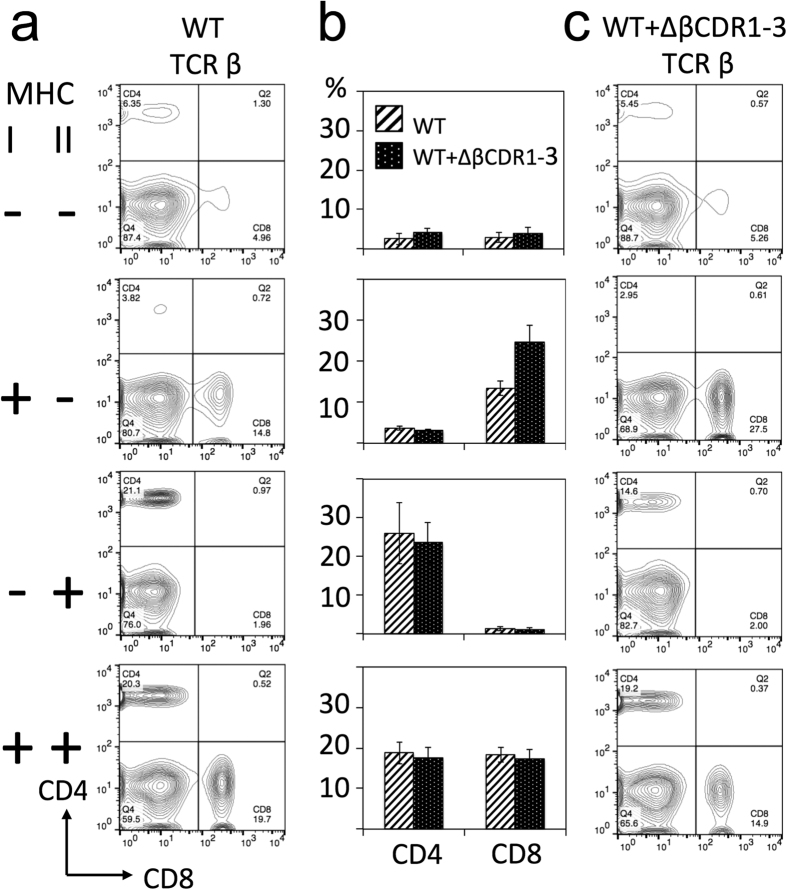
ΔβCDR1-3 expressing peripheral T cells are MHC restricted. Examples of splenic CD4 and CD8 T cell profiles of mice with WT TCR (**a**) and WT + ΔβCDR1-3 TCR (**c**). (**b**) Summary of data from multiple mice (n = 4–16 per group) showing percentages of splenic CD4 and CD8 T cells. Expression of MHC class I and II are indicated on the left.

**Figure 6:**
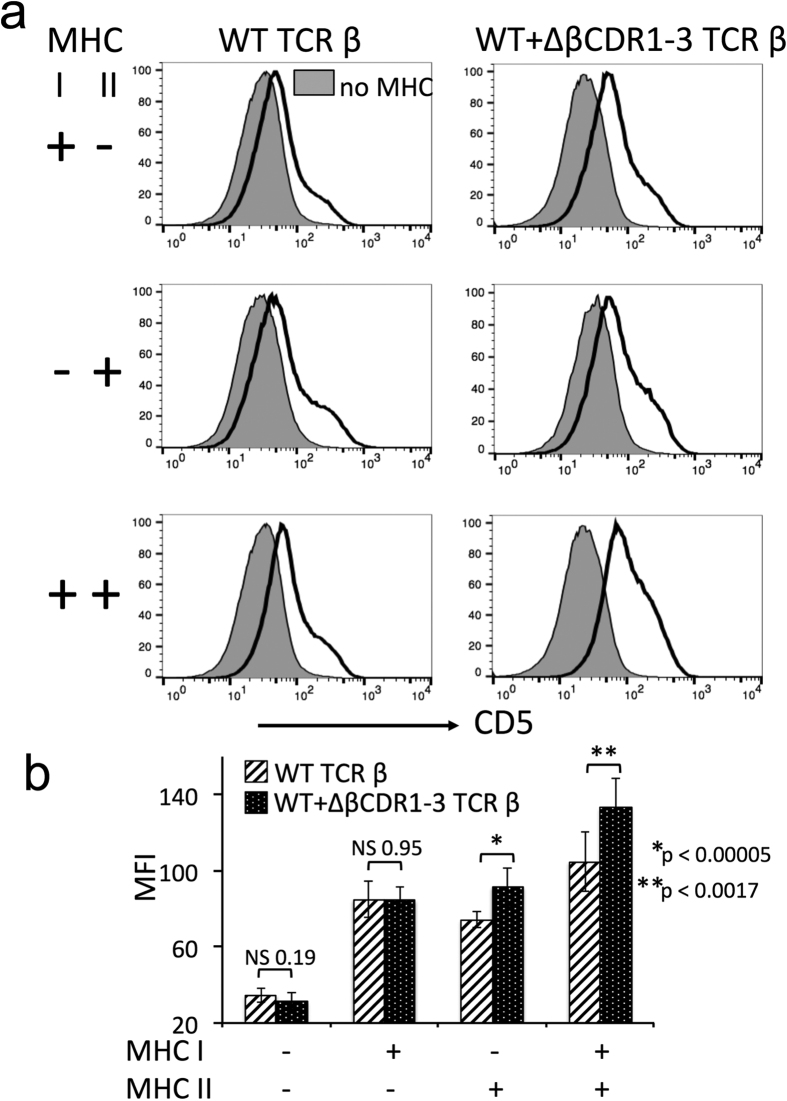
CD5 is not up-modulated by the ΔβCDR1-3 TCR in the absence of MHC. (**a**) Expression of CD5 on DP thymocytes in the absence of MHC I + II (filled plots) and presence of MHC I, MHC II and MHC I + II as indicated. Left plots are WT TCR and right plots are WT + ΔβCDR1-3. (**b**) Summary of CD5 mean fluorescence intensity on DP thymocytes (n = 4–16 per group).

Wild-type TCRs can engage with MHC and non-MHC ligands although, in the presence of the co-receptors, the latter are generally non-productive interactions^4^,^5^. We considered that loss of germline TCR structure could also promote recognition of non-MHC ligands, perhaps overriding co-receptor imposed MHC restriction and contributing to functional ligand engagement by the ΔβCDR1-3 TCR repertoire. To identify any such productive, non-MHC ligand engagement, the ΔβCDR1-3 mutant was crossed onto MHC deficient backgrounds. As previously shown^17^, in the absence of both MHC class I and II, few SP4/8 thymocytes (Fig. 4a,b) and peripheral CD4/8 T cells (Fig. 5a,b) are selected from wild-type TCR repertoires. Identical phenotypes were seen when the ΔβCDR1-3 mutant TCR-β was co-expressed (Figs 4b,c and 5b,c). In the absence of MHC I/II, CD5 expression on DP thymocytes is reduced reflecting the lack of TCR signalling^14^. Importantly, in this setting CD5 expression was expressed equivalently in the presence or absence of the ΔβCDR1-3 mutant (Fig. 6) excluding a role for non-MHC ligands in TCR signalling via ΔβCDR1-3 mutant TCRs. Individually, expression of MHC class I and class II gave rise exclusively to SP8/CD8 and SP4/CD4 populations respectively both in the absence and presence of the transgenic ΔβCDR1-3 mutant (Figs 4a–c and 5a–c). Despite ΔβCDR1-3 expression being lower than endogenous TCR in this cross, possibly due to co-expression with endogenous TCR (Supplementary Fig. S2), its presence significantly increased CD5 expression in the MHC class I^+^/II^+^ and MHC I^−^/II^+^ groups (Fig. 6). These data demonstrate that the ΔβCDR1-3 mutant CD4 and CD8 T cell repertoires formed by pairing with endogenous TCR-α chains are strictly MHC restricted.

## Germline TCR structure controls peptide cross-reactivity

**Figure 7:**
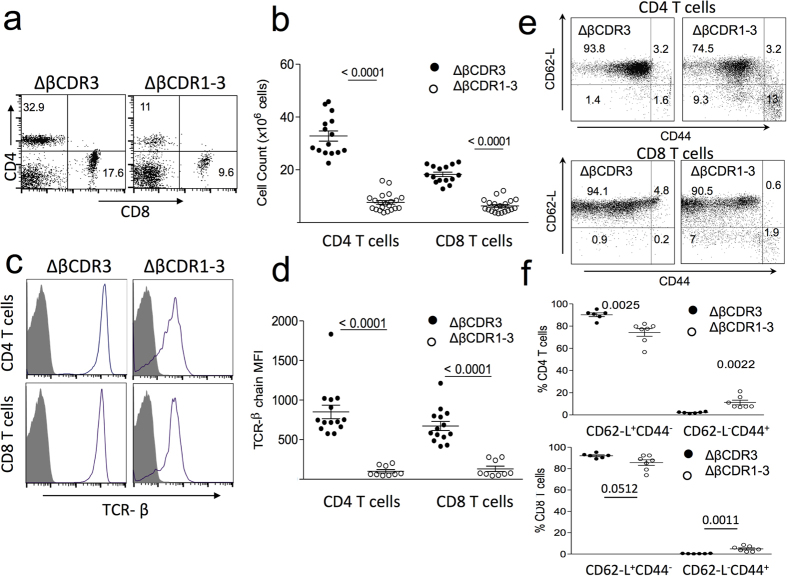
Phenotype of peripheral ΔβCDR3 and ΔβCDR1-3 T cells. (**a**) Examples of ΔβCDR3 and ΔβCDR1-3 spleen CD4 and CD8 T cell populations. (**b**) Summary of spleen CD4 and CD8 T cell numbers in ΔβCDR3 (n = 15) and ΔβCDR1-3 (n = 19) transgenic mice. (**c**) Example of TCR beta chain expression on ΔβCDR3 and ΔβCDR1-3 T cells. (**d**) Summary of TCR mean fluorescence intensity on ΔβCDR3 (n = 14) and ΔβCDR1-3 (n = 9) CD4 and CD8 T cells. (**e**) Expression CD62-L and CD44 were used to quantify naïve (CD62-L^+^, CD44^−^) and activated (CD62-L^−^, CD44^+^) populations of ΔβCDR3 and ΔβCDR1-3 CD4 and CD8 T cells. (**f**) Summary of percentages of naïve and activated populations in transgenic ΔβCDR3 (n = 6) and ΔβCDR1-3 mice (n = 7).

**Figure 8:**
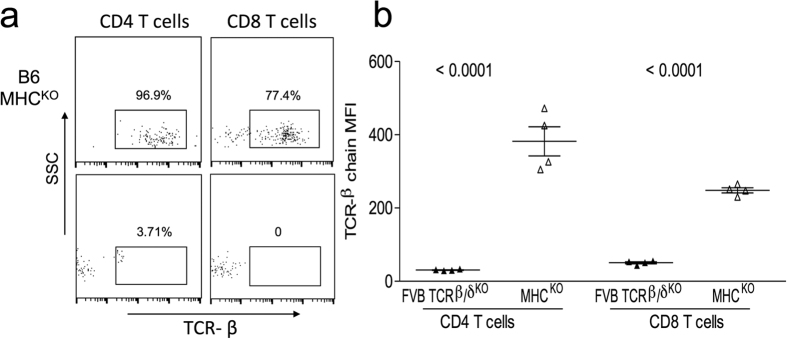
TCR on ΔβCDR1-3 T cells is down-modulated by MHC engagement. ΔβCDR1-3 splenocytes were adoptively transferred to C57BL/6 (MHC I/II deficient; n = 4) or FVB/N β/δ KO (MHC sufficient; n = 4) recipients. Both types of recipients are T cell deficient. After 14 days, TCR levels on peripheral blood CD4 and CD8 T cells were measured by flow cytometry. Examples of staining are shown in (**a**) and a summary of TCR-β MFI expression in (**b**).

Given the increased engagement of the ΔβCDR1-3 repertoire with MHC ligands during development, the impact on peripheral T cell-mediated immunity was assessed. The peripheral ΔβCDR1-3 T cell repertoire was distinct from the ΔβCDR3 in having smaller CD4 and CD8 T cell populations (Fig. 7a,b) reflecting the smaller thymic SP4 and SP8 populations (Fig. 2b). Further, surface TCR expression was reduced (Fig. 7c,d) and proportions of activated CD62-L^−^CD44^+^ CD4 and CD8 T cells were increased (Fig. 7e,f). Overall, this phenotype is consistent with constitutive TCR engagement promoting T cell differentiation. On-going engagement with MHC ligands as the cause of low surface TCR expression was investigated by adoptive transfer of ΔβCDR1-3 spleen cells into MHC I/II sufficient (FVB/N β/δ KO) or MHC I/II deficient (B6 MHC I/II KO) recipients. Consistent with this explanation, surface TCR was up-modulated in the absence of MHC but remained low in MHC sufficient recipients (Fig. 8).

**Figure 9:**
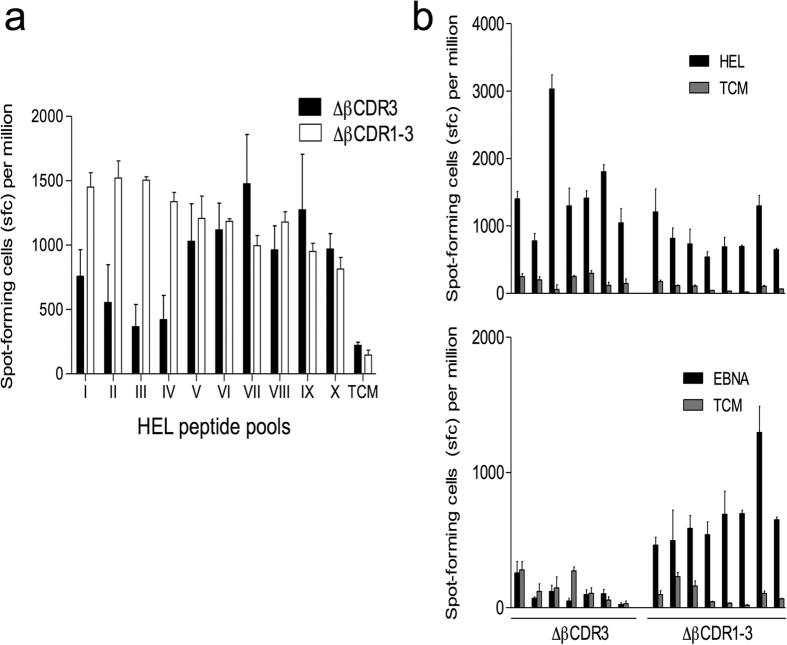
ΔβCDR1-3 T cells are highly cross-reactive. IFN-γ Elispot assays of responses by spleen cells harvested from mice immunised with HEL protein. Panel (a) shows that ΔβCDR1-3 mice (n = 4) respond robustly to a broader range of HEL-derived peptide pools (4–5 peptides) than ΔβCDR3 mice (n = 4). Panel (b) shows Elispot responses of HEL immunized mice [ΔβCDR3 (n = 7) and ΔβCDR1-3 mice (n = 8)] to whole HEL (upper) and whole EBNA1 peptide panel (lower). Each pair of bars represents a different mouse shown in the corresponding position in both charts. Negligible Elispot responses were seen in the absence of antigen (TCM; tissue culture medium only). Stimulation index (SI) for HEL and EBNA1 for each mouse was used to compare responses. For responses to HEL, ΔβCDR3 and ΔβCDR1-3 groups responded similarly (p = 0.93), while for responses to EBNA1, only the ΔβCDR1-3 group responded (p = 0.0078).

Antigen-specific T cell responses of ΔβCDR1-3 and control ΔβCDR3 transgenic mice were assessed by IFN-γ elispot following immunisation with hen egg lysozyme protein (HEL). Responses were assayed on pools of 4–5 HEL peptides showing ΔβCDR1-3 mice responded more robustly to 4/10 pools than control ΔβCDR3 mice (Fig. 9a). This observation prompted us to examine peptide cross-reactivity within the two repertoires. HEL immunised mice were assessed for recognition of peptides from the unrelated protein Epstein-Barr virus nuclear antigen 1 (EBNA1). Both strains responded similarly to whole HEL protein (Fig. 9b upper). Strikingly, HEL immunised ΔβCDR1-3, but not ΔβCDR3 mutant mice, also responded to the EBNA1 peptide pool (Fig. 9b lower). These data demonstrate that TCR cross-reactivity to is regulated by germline TCR structure.

## Discussion

Our findings provide further evidence that germline CDRs do not hardwire the TCR for recognition of MHC ligands^4^,^5^,^9^,^16^,^18^,^19^. Rather, we show they have the opposite function of moderating engagement with MHC ligands. The ΔβCDR1-3 mutant studied here has germline CDRs with simpler, flexible loop structures through substitution of larger, complex amino-acid side-chains (H, Y, R, N, Q) for glycine and alanine. We hypothesise that the modified loops reduce steric hindrance effects when engaging with MHC ligands increasing the average affinity of the interface accounting for the increase in CD5 expression across the DP pre-selection thymocyte repertoire and the increased TCR^hi^ and CD69^hi^ components (Fig. 3, Supplementary Fig. S1). Conversely, the more complex and rigid^20^ wild-type structures will less frequently find compatible interfaces and thus enrich the repertoire with T cells that generally engage MHC ligands within a lower affinity range. Indeed the germline β CDR1 and 2 are known to undergo the smallest conformational changes on binding MHC ligands^20^. The moderating influence of germline structure on MHC engagement can likely be achieved in many ways consistent with the sequence diversity of CDR1 and 2 across the α and β variable segment repertoires^9^ which results in substantial combinatorial complexity.

As thymic positive selection proceeds at extremely low affinities^21^, a degree of steric hindrance may offer an efficient solution to achieving optimal engagement across the set of highly polymorphic MHC I and II ligands. Indeed, chimeric TCR-β chains with γδ T cell lineage, Ig heavy and Ig light chain CDRs are capable of productively engaging with MHC I and II^9^,^22^ (Fig. 1).

In the context of wild-type germline structure, single amino-acid substitutions, in particular tyrosine, can diminish MHC engagement^23^. However, the same residues are also important for engagement with non-MHC ligands^16^ and are enriched within Ig germline CDRs^24^ suggesting generic properties such as versatile Van der Waals interactions are employed to contribute to interface formation. Strikingly, loss of germline TCR structure did not compromise MHC restriction highlighting the efficiency of co-receptor imposed ligand bias^18^.

The majority of TCR:MHC:peptide co-crystals show the TCR engages MHC ligands with a fixed orientation and constrained geometry^25^ which, at least for MHC class I, can promote productive signalling^26^. Our findings do not support the idea that germline TCR structure is crucial for directing docking topology as functional engagement with MHC was increased in the absence of germline structure. Indeed, two TCRs derived from human induced T regulatory cells bound MHC class II with reversed polarity^19^. A mechanistic link between docking topology and signalling competence has been proposed to involve appropriate positioning of co-receptor bound lck with CD3 ITAMs^27^. Whether the modified ΔβCDR1-3 germline CDR loops influence docking topology is unclear but we may infer that the germline CDRs remain MHC-centric^28^ rather than peptide-centric as the ΔβCDR1-3 peripheral repertoire is highly peptide cross-reactive, which would not be expected if self-peptides were strongly engaged by the germline CDRs.

We propose that increased engagement of MHC by ΔβCDR1-3 TCRs shifts the pre-selection repertoire towards negative selection (Figs 2 and 3) favoring survival of DP thymocytes whose TCR CDR3 loops make only limited contact with self-peptide. In similar fashion, MHC-centric ΔβCDR1-3 TCRs on peripheral T cells would reduce the contribution required from non-self peptide to reach the threshold for T cell activation accounting for their increased peptide cross-reactivity (Fig. 9). On the other hand, wild-type CDR structures, by engaging less well with MHC, place greater emphasis on CDR3 engagement with peptide resulting in lower peptide cross-reactivity. The T cell compartment must be able to recognise virtually any peptide, this is orders of magnitude greater than the number of T cell clones in an individual. TCR cross-reactivity is therefore an essential feature of the T cell repertoire^29^; indeed, peptide cross-reactivity of several TCRs has been estimated to be typically >10^6^ 28,30,31. We conclude that germline structure governs this crucial aspect of the TCR repertoire by ‘fine-tuning’ TCR engagement with MHC ligands. In WT TCRs, cross-reactivity is mostly due to tolerance of peptide substitutions to residues outside of the TCR interface and relatively conservative changes to residues that contact the CDR3 loops whilst maintaining germline CDR-MHC docking^28^. ΔβCDR1-3 germline CDRs with simpler, flexible loop structures will have broader docking options providing a mechanism to further increase cross-reactivity.

In summary, the BCR and αβTCR appear to use their germline regions in a functionally analogous manner to achieve their distinct strategies of antigen recognition. Co-receptor mediated sequestration of lck focuses the αβTCR repertoire onto MHC ligands allowing the germline regions to ‘fine-tune’ the affinity of this engagement and regulate peptide cross-reactivity.

Although both arms of adaptive immunity evolved in early jawed vertebrates with primitive extant species (cartilaginous fish) having the key components (RAG 1/2, BCR, TCR, MHC I/II, CD4 and CD8), their evolutionary relationship is unclear. We speculate that ‘antibody-like’ recognition of ligand by receptors of both lineages may have allowed variable segments to be more easily ‘co-opted’ to function as part of the alternative strategy of antigen recognition simplifying the early evolutionary path of adaptive immunity.

## Methods

### Mice

Mice were housed under specific pathogen-free conditions at Imperial College London. All experimental protocols were approved by the Institutional Animal Welfare and Ethical Review Committee and the Home Office and were performed in accordance with the relevant guidelines and regulations. WT FVB/N (H2^q^) mice were obtained from Harlan Laboratories, Loughborough, UK. FVB/N TCR-β/δ^KO^ were a kind gift from Prof. Daniel Pennington. C57BL/6 MHC class I, II deficient mice^32^ were a kind gift from Prof. Matthias Merkenschlager. TCR-β chain variants were synthesised (Mr Gene, Regensburg, Germany). Genetically modified mice (retrogenic and transgenic) were produced directly on an FVB/N TCR-β/δ^KO^ background. Disruption at the *trb* locus prevents expression of endogenous TCR-β chains and αβ T cell development. Developing αβ T cells must use the exogenous TCR-β chain^9^,^11^. For retrogenic expression, TCR-β chains were cloned into the pMigR1 retroviral vector and transduced into FVB/N TCR-β/δ^KO^ haematopoietic stems cells (HSC) which were then injected intravenously into irradiated (600 Rad) FVB/N TCR-β/δ^KO^ recipients as previously described^9^,^11^. For conventional transgenic production, the T cell specific expression vector pVA-hCD2^33^ was used which gives position independent expression directed by the CD2 LCR. For each transgene, two founders were produced which had indistinguishable phenotypes.

To breed the ΔβCDR1-3 transgenic strain onto MHC deficient backgrounds (MHC I^+^ II^+^; MHC I^+^ II^−^; MHC I^−^ II^+^; MHC I^−^ II^−^) it was back-crossed with MHC class I, II deficient mice (H2^b^) and the offspring typed by flow cytometry for MHC I, MHC II and TRBV16 for the transgenic TCR.

### Immunisation

6 week old female mice were immunised (s.c.) by injection of 200 μl of HEL (final concentration 7 nM) (Sigma, Poole, UK) emulsified in complete Freund’s adjuvant (CFA) and boosted after 7 days with HEL emulsified in incomplete Freund’s adjuvant (IFA). Mice were sacrificed after a further 7 days.

### IFN-γ ELISpot immunoassay

An Elispot kit (Genprobe-Diaclone, Besançon, France) was used in accordance with the manufacturer’s directions. In brief, PVDF 96-well plates (Millipore, Watford, UK) were activated with 70% ethanol then coated with 100 μl of 10 μg/ml of capture antibody overnight (4 °C) then washed 6X with sterile PBS. Plates were then blocked with PBS/2% powdered milk and washed. 10^5^ responder cells were added to each well along with antigen and incubated overnight at 37 °C/5% CO_2_. Plates were then washed 6X with 100 μl PBS/0.1% Tween-20 and the biotinylated detection antibody added. After 2 hrs, the plates were washed 6X as before. 100 μl of streptavidin-alkaline phosphatase was then added (1 hr/37 °C) and the plates developed. Spot forming cells were visualised using the AID ELISpot plate reader (AID Diagnostika, Germany). Antigens: HEL (Sigma, Poole, UK); HEL peptide pool of 24 15mers with 10 amino acid overlap (GL Biochem, Shanghai, China) and EBV EBNA1 peptide pool (JPT Peptide Technologies, Berlin, Germany) with 158 15mer peptides with 11 aa overlap.

### Cell preparation

Tissues were harvested from 5–8 week old wild-type transgenic mice and from retrogenic mice 5–7 weeks after reconstitution. Cell suspensions were prepared using a sterile cell strainer and washed twice in 2 ml of phosphate-buffered saline (PBS). Blood and spleen preparations suspended for 30 minutes at room temperature in red blood cell lysis buffer (Qiagen, Hilden, Germany) to lyse red blood cells, centrifuged and resuspended as appropriate for the procedure.

### Flow Cytometry

Cell suspensions were stained with fluorochrome-conjugated monoclonal antibodies against CD4, CD8, TCR-β constant region, TRBV16, CD44, CD62-L, CD5, CD69, MHC I, MHC II (all from BD Biosciences). Samples were incubated for twenty minutes at room temperature, washed in 2 ml of PBS and kept at 4 °C until acquisition on a BD FACSCalibur^TM^ flow cytometer using the Cellquest^TM^ Software (BD). A live lymphocyte gate was applied to all samples. Further gating was done on CD4^+^ and CD8^+^ as appropriate. Data analysis was performed on FlowJo version 5.7 (Tree Star Inc, US).

### Statistics

Inter-group variations were analysed by two-tailed, unpaired t-tests using GraphPad Prism 5. Results are shown graphically as mean and standard error of the mean (SEM). P values are given for 95% confidence intervals.

## Additional Information

**How to cite this article**: Attaf, M. *et al*. αβ T cell receptor germline CDR regions moderate contact with MHC ligands and regulate peptide cross-reactivity. *Sci. Rep.* **6**, 35006; doi: 10.1038/srep35006 (2016).

## Supplementary Material

Supplementary Information

## References

[b1] Veillette A., Bookman M. A., Horak E. M. & Bolen J. B. The CD4 and CD8 T cell surface antigens are associated with the internal membrane tyrosine-protein kinase p56 lck. Cell 55, 301–308 (1988). 3262426 10.1016/0092-8674(88)90053-0

[b2] Veillette A., Bookman M. A., Horak E. M., Samelson L. E. & Bolen J. B. Signal transduction through the CD4 receptor involves the activation of the internal membrane tyrosine-protein kinase p56lck. Nature 338, 257–259 (1989). 2784195 10.1038/338257a0

[b3] Chalupny N. J., Ledbetter J. A. & Kavathas P. Association of CD8 with p56lck is required for early T cell signaling events. EMBO J. 10, 1201–1207 (1991). 1902413 10.1002/j.1460-2075.1991.tb08061.x PMC452774

[b4] Van Laethem F. . Deletion of CD4 and CD8 coreceptors permits generation of αβT cells that recognize antigens independently of the MHC. Immunity 27, 735–750 (2007). 18023370 10.1016/j.immuni.2007.10.007

[b5] Van Laethem F. . Lck availability during thymic selection determines the recognition specificity of the T cell repertoire. Cell 154, 1326–1341 (2013). 24034254 10.1016/j.cell.2013.08.009 PMC3792650

[b6] Li X. L., Teng M. K., Reinherz E. L. & Wang J. H. Strict Major Histocompatibility Complex Molecule Class-Specific Binding by Co-Receptors Enforces MHC-Restricted αβ TCR Recognition during T Lineage Subset Commitment. Front Immunol 4, 383 (2013). 24319443 10.3389/fimmu.2013.00383 PMC3837227

[b7] Marrack P., Scott-Browne J. P., Dai S., Gapin L. & Kappler J. W. Evolutionarily conserved amino acids in TCR V regions and MHC control their interaction. Annu. Rev. Immunol. 26, 171–203 (2008). 18304006 10.1146/annurev.immunol.26.021607.090421 PMC3164820

[b8] Garcia K. C., Adams J. J., Feng D. & Ely L. K. The molecular basis of TCR germline bias for MHC is surprisingly simple. Nature Immunology 10, 143–147 (2009). 19148199 10.1038/ni.f.219 PMC3982143

[b9] Holland S. J. . The T-cell receptor is not hardwired to engage MHC ligands. Proc. Natl. Acad. Sci. USA 109, 3111–3118 (2012). 10.1073/pnas.1210882109 PMC3494948 23077253

[b10] Bartok I. . T cell receptor CDR3 loops influence αβ pairing. Mol. Immunol. 47, 1613–1618 (2010). 20189651 10.1016/j.molimm.2010.01.012

[b11] Furmanski A. L. . Public T cell receptor β-chains are not advantaged during positive selection. J. Immunol. 180, 1029–1039 (2008). 18178843 10.4049/jimmunol.180.2.1029

[b12] Al-Lazikani B., Lesk A. M. & Chothi C. Canonical structures for the hypervariable regions of T cell αβ receptors. J. Mol. Biol. 295, 979–995 (2000). 10656805 10.1006/jmbi.1999.3358

[b13] Stritesky G. L. . Murine thymic selection quantified using a unique method to capture deleted T cells. Proc. Natl. Acad. Sci. USA 110, 4679–4684 (2013). 23487759 10.1073/pnas.1217532110 PMC3606987

[b14] Azzam H. S. . CD5 expression is developmentally regulated by T cell receptor (TCR) signals and TCR avidity. J. Exp. Med. 188, 2301–2311 (1988). 10.1084/jem.188.12.2301 PMC2212429 9858516

[b15] Huesmann M., Scott B., Kisielow P. & von Boehmer H. Kinetics and efficacy of positive selection in the thymus of normal and T cell receptor transgenic mice. Cell 66, 533–540 (1991). 1868548 10.1016/0092-8674(81)90016-7

[b16] Tikhonova A. N. . αβ T cell receptors that do not undergo major histocompatibility complex-specific thymic selection possess antibody-like recognition specificities. Immunity 36, 79–91 (2012). 22209676 10.1016/j.immuni.2011.11.013 PMC3268851

[b17] Brändle D., Müller S., Müller C., Hengartner H. & Pircher H. Regulation of RAG‐1 and CD69 expression in the thymus during positive and negative selection. European Journal of Immunology. 24, 145–151 (1994). 8020549 10.1002/eji.1830240122

[b18] Van Laethem F., Tikhonova A. N. & Singer A. MHC restriction is imposed on a diverse T cell receptor repertoire by CD4 and CD8 co-receptors during thymic selection. Trends. Immunol. 33, 437–441 (2012). 22771139 10.1016/j.it.2012.05.006 PMC3427466

[b19] Beringer D. X. . T cell receptor reversed polarity recognition of a self-antigen major histocompatibility complex. Nat. immunol. 16, 1153–1161 (2015). 26437244 10.1038/ni.3271

[b20] Armstrong K. M., Piepenbrink K. H. & Baker B. M. Conformational changes and flexibility in T-cell receptor recognition of peptide-MHC complexes. Biochem. J. 415, 183–196 (2008). 18800968 10.1042/BJ20080850 PMC2782316

[b21] Juang J. . Peptide-MHC heterodimers show that thymic positive selection requires a more restricted set of self-peptides than negative selection. J. Exp. Med. 207, 1223–1234 (2010). 20457759 10.1084/jem.20092170 PMC2882826

[b22] Bowen S., Sun P., Livak F., Sharrow S. & Hodes R. J. A novel T cell subset with trans-rearranged Vγ-Cβ TCRs shows Vβ expression is dispensable for lineage choice and MHC restriction. J. Immunol. 192, 169–177 (2014). 24307734 10.4049/jimmunol.1302398 PMC3872174

[b23] Scott-Browne J. P., White J., Kappler J. W., Gapin L. & Marrack P. Germline-encoded amino acids in the alpha beta T-cell receptor control thymic selection. Nature 458, 1043–1046 (2009). 19262510 10.1038/nature07812 PMC2679808

[b24] Ofran Y., Schlessinger A. & Rost B. Automated identification of complementarity determining regions (CDRs) reveals peculiar characteristics of CDRs and B cell epitopes. J. Immunol. 181, 6230–6235 (2008). 18941213 10.4049/jimmunol.181.9.6230

[b25] Collins E. J. & Riddle D. S. TCR-MHC docking orientation: natural selection, or thymic selection? Immunol. Res. 41, 267–294 (2008). 18726714 10.1007/s12026-008-8040-2

[b26] Adams J. J. . T cell receptor signaling is limited by docking geometry to peptide-major histocompatibility complex. Immunity 35, 681–693 (2011). 22101157 10.1016/j.immuni.2011.09.013 PMC3253265

[b27] Rangarajan S. & Mariuzza R. A. T cell receptor bias for MHC: co-evolution or co-receptors? Cell. Mol. Life Sci. 71, 3059–3068 (2014). 24633202 10.1007/s00018-014-1600-9 PMC11113676

[b28] Adams J. J. . Structural interplay between germline interactions and adaptive recognition determines the bandwidth of TCR-peptide-MHC cross-reactivity. Nat Immunol 17, 87–94 (2016). 26523866 10.1038/ni.3310 PMC4684756

[b29] Mason D. A very high level of crossreactivity is an essential feature of the T-cell receptor. Immunology today 19, 395–404 (1998). 9745202 10.1016/s0167-5699(98)01299-7

[b30] Maynard J. . Structure of an autoimmune T cell receptor complexed with class II peptide-MHC: insights into MHC bias and antigen specificity. Immunity 22, 81–92 (2005). 15664161 10.1016/j.immuni.2004.11.015

[b31] Wooldridge L. . A single autoimmune T cell receptor recognizes more than a million different peptides. Journal of Biological Chemistry 287, 1168–1177 (2012). 22102287 10.1074/jbc.M111.289488 PMC3256900

[b32] Chan S. H., Cosgrove D., Waltzinger C., Benoist C. & Mathis D. Another view of the selective model of thymocyte selection. Cell 73, 225–236 (1993). 8097430 10.1016/0092-8674(93)90225-f

[b33] Zhumabekov T., Corbella P., Tolaini M. & Kioussis D. Improved version of a human CD2 minigene based vector for T cell-specific expression in transgenic mice. J. Immunol. Methods 185, 133–140 (1995). 7665895 10.1016/0022-1759(95)00124-s

