# Supplementary material for: αβ T cell receptor germline CDR regions moderate contact with MHC ligands and regulate peptide cross-reactivity
Source: Sci Rep. 2016 Oct 24;6:35006. doi: 10.1038/srep35006 (PMC5075794; doi:10.1038/srep35006)
Supplement: Supplementary Information [file srep35006-s1.pdf]

Supplementary information

αβ T cell receptor germline CDR regions moderate contact with MHC ligands and regulate peptide cross-reactivity

Meriem Attaf[1, 2], Stephan J. Holland[1, 3], Istvan Bartok[1, 4], Julian Dyson[1, 4, *]

Affiliations:

[1]Section of Molecular Immunology, Department of Medicine, Imperial College London, Du Cane Road, London, W12 0NN, UK

[2]Present address; Division of Infection and Immunity, Cardiff University School of Medicine, Cardiff, CF14 4XN, UK.

[3]Present address; Department of Developmental Immunology, Max Planck Institute of Immunobiology and Epigenetics, Stübeweg 51, D-79108 Freiburg, Germany.

[4]These authors contributed equally.

[*]Present address; Adaptimmune Ltd, 101 Park Drive, Milton Park, Abingdon, Oxfordshire, OX14 4RY, UK. Corresponding author; email julian.dyson@adaptimmune.com

Supplementary Figure S1

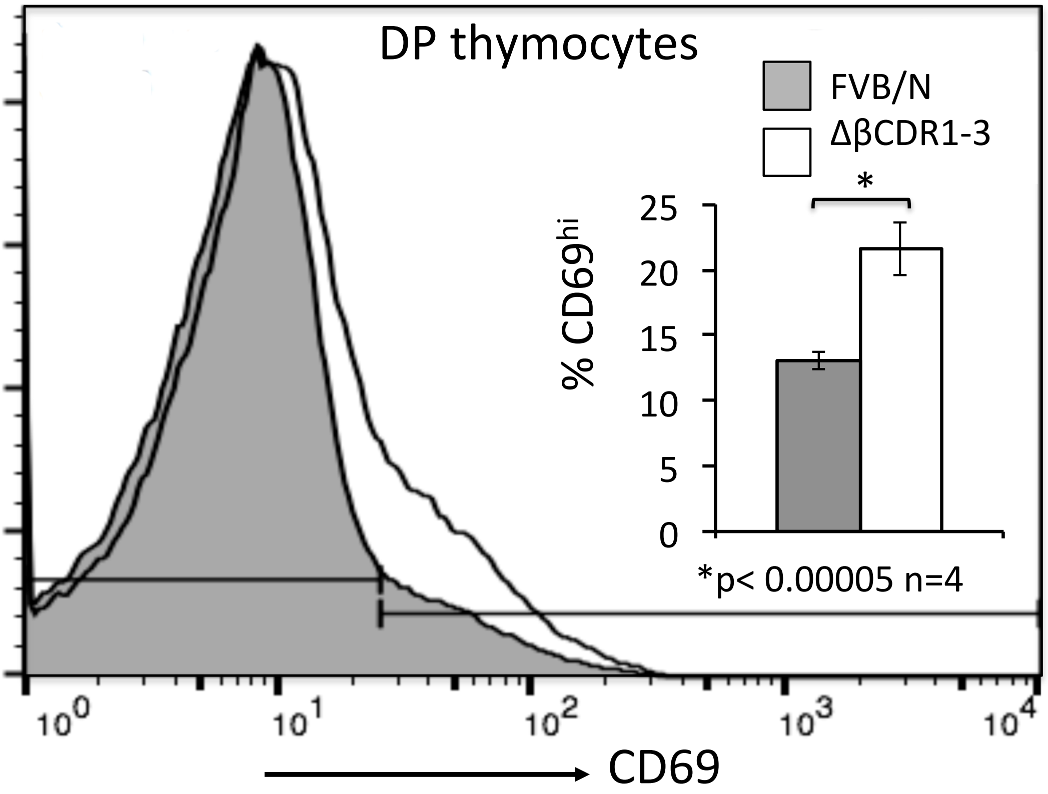

CD69 expression on ΔβCDR1-3 and WT DP thymocytes

CD69 expression on ΔβCDR1-3 DP thymocytes is significantly higher than on WT FVB/N DP thymocytes. One example of CD5 expression is shown. The insert shows summary of CD5 expression (n=4)

Supplementary Figure S2

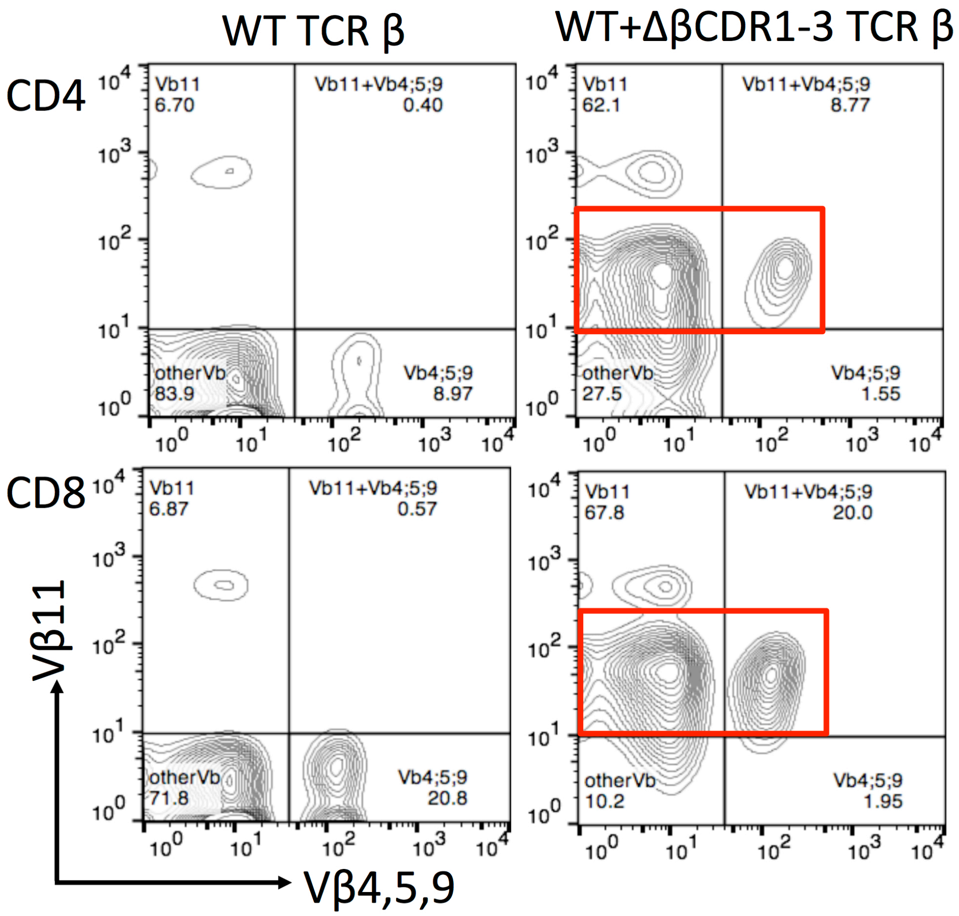

ΔβCDR1-3 transgenic TCR is expressed in the presence of a WT TCR repertoire

Plots show expression of Vβ11 (Y axis) and Vβ4, 5, 9 (X axis) on gated CD4 (upper plots) and CD8 T cells (lower plots). Left plots (WT TCRβ) show small and $(V\beta4, 5, 9)^+$ CD4 and CD8 populations. Right plots (WT+ΔβCDR1-3 TCRβ) show that most CD4 and CD8 T cells are $V\beta11^+$ (boxed region) representing the transgenic chain. The transgenic Vβ11 chain is co-expressed on the $V\beta4, 5, 9)^+$ populations.
